# Cross-Recognition of SARS-CoV-2 B-Cell Epitopes with Other Betacoronavirus Nucleoproteins

**DOI:** 10.3390/ijms23062977

**Published:** 2022-03-10

**Authors:** Ana Tajuelo, Mireia López-Siles, Vicente Más, Pilar Pérez-Romero, José María Aguado, Verónica Briz, Michael J. McConnell, Antonio J. Martín-Galiano, Daniel López

**Affiliations:** 1 Centro Nacional de Microbiología, Instituto de Salud Carlos III, 28220 Majadahonda, Madrid, Spain; atajuelo@isciii.es (A.T.); mireia.lopez@isciii.es (M.L.-S.); vmas@isciii.es (V.M.); mpperez@isciii.es (P.P.-R.); vbriz@isciii.es (V.B.); mgaliano@isciii.es (A.J.M.-G.); 2 Hospital Universitario “12 de Octubre”, 28041 Madrid, Spain; jmaguado@h12.es

**Keywords:** emerging disease, B cells, antibodies, cross-reactivity, SARS-CoV-2, humoral response

## Abstract

The B and T lymphocytes of the adaptive immune system are important for the control of most viral infections, including COVID-19. Identification of epitopes recognized by these cells is fundamental for understanding how the immune system detects and removes pathogens, and for antiviral vaccine design. Intriguingly, several cross-reactive T lymphocyte epitopes from SARS-CoV-2 with other betacoronaviruses responsible for the common cold have been identified. In addition, antibodies that cross-recognize the spike protein, but not the nucleoprotein (N protein), from different betacoronavirus have also been reported. Using a consensus of eight bioinformatic methods for predicting B-cell epitopes and the collection of experimentally detected epitopes for SARS-CoV and SARS-CoV-2, we identified four surface-exposed, conserved, and hypothetical antigenic regions that are exclusive of the N protein. These regions were analyzed using ELISA assays with two cohorts: SARS-CoV-2 infected patients and pre-COVID-19 samples. Here we describe four epitopes from SARS-CoV-2 N protein that are recognized by the humoral response from multiple individuals infected with COVID-19, and are conserved in other human coronaviruses. Three of these linear surface-exposed sequences and their peptide homologs in SARS-CoV-2 and HCoV-OC43 were also recognized by antibodies from pre-COVID-19 serum samples, indicating cross-reactivity of antibodies against coronavirus N proteins. Different conserved human coronaviruses (HCoVs) cross-reactive B epitopes against SARS-CoV-2 N protein are detected in a significant fraction of individuals not exposed to this pandemic virus. These results have potential clinical implications.

## 1. Introduction

Immunological cross-responses between the severe acute respiratory syndrome coronavirus 2 (SARS-CoV-2) and seasonal human coronaviruses (HCoVs) have raised the interest of the scientific community. CD4^+^ and CD8^+^ co-stimulating epitopes of SARS-CoV-2 and homologous proteins in other HCoVs that produce seasonal infections were discovered early during the pandemic and associated with partial protection [1,2,3,4]. Equivalent analyses for the humoral response, which was first defined for the coronavirus causing the 2003 pandemic [5], have also been carried out [6,7,8]. These studies have mainly been focused on the spike protein, for which cross-reactivity developed for SARS-CoV-2 to the HCoV homolog is rare and weak [6,9,10,11,12,13], and very likely limited to some remote similarity between discontinuous epitopes. Early in these pandemics it was noticed that nucleocapsid cross-reactivity to other betacoronaviruses is a usual source of false positives in antigenic tests [14]. This finding has promoted the identification of three and one linear B-cell epitopes from the spike and N viral proteins, respectively, with high discriminatory power [1].

Fundamental aspects of the humoral memory response against SARS-CoV-2 remain to be revealed [15]. The humoral response, in contrast to the cellular response, is associated with a poor prognosis and potential antibody-dependent immunopathogenesis [16]. While asymptomatic individuals show low anti-nucleocapside protein antibody titers, their T-cell responses are strong [17]. In particular, the lack of early serological skew from the nucleocapsid to the spike protein is further associated with more severe infection [18]. However, affinity matured humoral responses are protective after re-infection and, indeed, the pursued goal of current vaccines. This understanding can result in improved specificity in diagnosis and prophylactic efficacy that, ultimately, ameliorate the effects of the disease. Multiple B-cell epitopes from SARS-CoV and SARS-CoV-2 have been experimentally validated [19] and included in the Immune Epitope Database (IEDB), a central repository that stores, catalogs, and assists in the prediction and analysis of epitopes [20]. Together with computational predictive tools, these data can be used to study the cross-reactive humoral response in coronaviruses.

Similar to other betacoronaviruses, the SARS-CoV-2 capsid consists of four structural proteins accessible to antibodies (Abs). The small envelope (E) and the membrane (M) proteins are involved in envelope formation [21]. The polymeric nucleocapsid (N) protein plays roles related to the viral RNA, including the packaging and the regulation of synthesis, and may be involved in overcoming host defense by suppressing RNA interference mechanisms [22]. Homologs of this protein from other zoonotic coronaviruses were identified as highly immunogenic with antigenic sites throughout the entire sequence [23,24,25]. Moreover, betacoronavirus nucleocapsid protein is detectable in clinical samples after the onset of symptoms and thus, serves as an early diagnostic marker [26]. Lastly, the spike (S) protein contains the receptor-binding domain (RBD), which is required for viral attachment to the cell surface ACE2 receptor of lung epithelia and other host cells. This S protein is the major target for current vaccine development [27].

For antibody-based therapeutics, rapid diagnostic tests, development of vaccines, and other clinical approaches against COVID-19, the identification of SARS-CoV-2 B-cell epitopes, which are the key elements of the protective humoral immune response, is fundamental. In addition, characterization of the viral sequences recognized by SARS-CoV-2-specific Abs can also contribute to identifying the mutational changes relevant for the ability of the immune response to provide cross protection against betacoronaviruses.

Whether humoral cross-responses between SARS-CoV-2 and HCoVs are hardly anecdotal or may actually exert a clinical impact remain to be examined in depth. In this study, universal candidate dominant B-cell epitopes the potential cross-reactive humoral response for SARS-CoV-2 have been explicitly assessed using a computational-experimental approach. For that, universal B-cell dominant candidate epitopes conserved in betacoronaviruses have been selected in a pre-deterministic manner using immunoinformatic tools and IEDB data. Reactivity with antisera from pre-COVID-19 samples indicates that four sequences within the N protein may behave as universal epitopes.

## 2. Results

### 2.1. Mapping of Conserved B-Cell Antigenic Regions between Structural Proteins of SARS-CoV-2 and Other Coronaviruses

**Figure 1:**
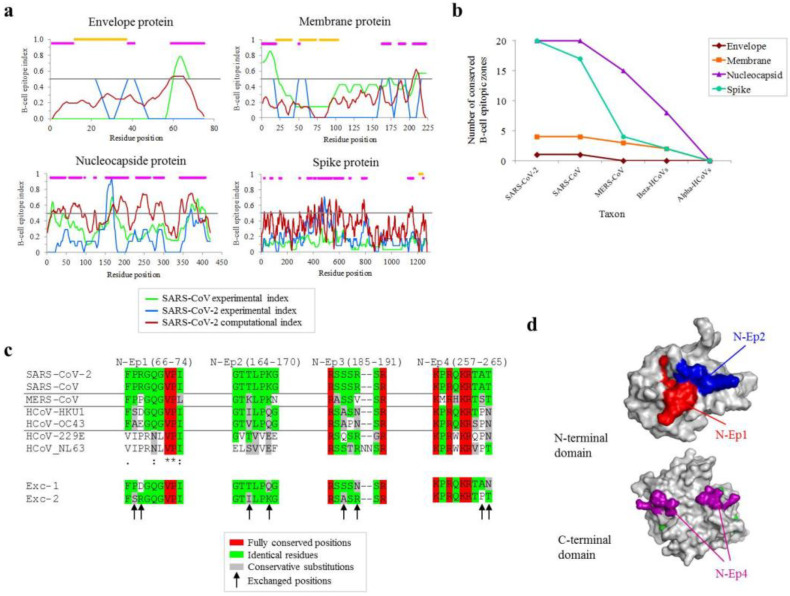
Conservation of B-cell epitope zones at different taxonomical ranges. (**a**) Consensus B-cell epitope in the four virion proteins after computational prediction (red line), and experimental validation in SARS-CoV and SARS-CoV-2 applying a window of seven residues. The line indicates the 0.5 threshold applied to select epitopes. Consensus epitopes are in fuchsia. Transmembrane sections are in orange. (**b**) Taxonomical stepwise conservation of virion betacoronavirus antigens: number of conserved positions globally in the whole alignment (up to 15mers, split into proportional sections if more) extends ≥ 7 positions over 65% similarity and three identical residues, without indels. (**c**) Alignments of wild-type sequences and proposed consensus peptide sequences for the four most conserved epitopes in the nucleocapside protein. The degree of conservation is color-ranked (see inset legend). Shuffled positions in exchanged epitopes (Exc-1 and Exc-2) are shown (arrow). (**d**) Structural mapping of conserved epitopes. N-t nucleotide-binding (above) and C-t dimerization (below) domains are shown.

Predicted B-cell antigenic regions in the four SARS-CoV-2 structural proteins were determined by the consensus of eight bioinformatic methods. Additionally, the collection of experimentally detected epitopes for SARS-CoV and SARS-CoV-2 stored in the IEDB was included. Consensus of these predicted and experimental analyses revealed different epitope-enriched regions in the four proteins (Figure 1a, fuchsia bars). Spans of ≥7 non-transmembrane residues showing scores of ≥0.5 for any of the three strategies (see Methods/Legend) were considered B-cell antigenic. These B-cell epitopic regions were numerous in the N and the S proteins, and particularly longer in the former. The conservation of the dense epitope zones in other coronaviruses was estimated. Given the maximal lengths of B-cell epitopes, long zones in the N protein were split into 15mers for this analysis. Most epitope zones were deemed conserved (threshold: ≥65% similarity in ≥7 residues, at least three identical residues and no indels) in SARS-CoV but sharply declined as taxonomic distance was incremented to Middle East Respiratory Syndrome-related coronavirus (MERS-CoV) while only a few zones (N: 7; M: 2; S: 3) were conserved at the betacoronavirus stratum, which included seasonal common cold strains (Figure 1b). None were conserved at the alphacoronavirus level according to these parameters. From the 12 zones positive at the betacoronavirus level, however, only four with ≥7 residues from N protein showed identity for all positions except from two in the closest seasonal virus HCoV-HKU1 (Figure 1c). Residues in these positions in SARS-CoV2 and HCoV-HKU1 were swapped to create two chimeric exchange peptides (Exc-1 and Exc-2) for further experiments (Figure 1c, see asterisks). Three out of the four selected peptides located in structurally resolved fragments of the N protein surface: N-Ep1 (red), N-Ep2 (blue), and N-Ep4 (fuchsia). In contrast, N-Ep3 sequence is located in an unstructured inter-domain linker region and, thus, its structure could not be determined by crystallography. In summary, all these regions are very likely accessible for antibody binding (Figure 1d). Altogether, this analysis identified four surface-exposed, conserved, and hypothetical antigenic regions that are exclusive of the N protein, and different than those previously described in [1].

### 2.2. Serologic Reactivity of Anti-S and -N IgG Abs in a Cohort of Healthcare Workers Affected by COVID-19

**Figure 2:**
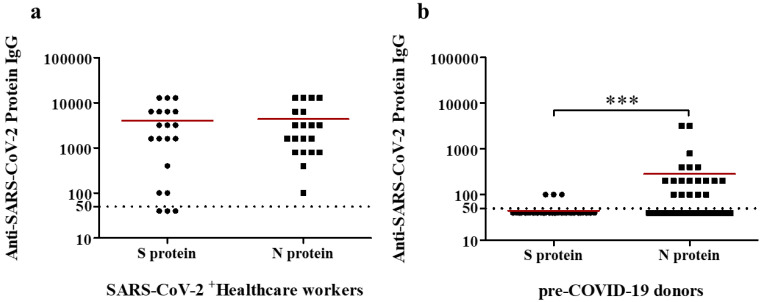
Analysis of serum IgG antibodies against SARS-CoV-2 S and N proteins in healthcare workers with COVID-19 and pre-COVID-19 samples determined by ELISA assay. Cutoff values to determine positive (above), and negative (below dashed line) samples is indicated. Dots and squares represent anti-SARS-CoV-2 S and N protein IgGs, respectively, from healthcare workers affected by COVID-19 (**a**) or pre-pandemic samples (**b**). Means (red lines) and significant P values (***, *p* < 0.001 Mann Whitney test) are indicated.

Antibody responses against SARS-CoV-2 in 21 subjects affected by COVID-19 were determined by the presence of total anti-SARS-CoV-2 IgG Abs by ELISA. Little to no IgG response against SARS-CoV-2 S protein were detected in 3 and 2 of the healthcare workers affected by COVID-19, respectively (Figure 2a). Antibody titer in other patient was 400, while the other 14 ranged between 1600 and 12,800 in the ELISA assay (Figure 2a). In addition, the presence of anti-SARS-CoV-2 IgG Abs targeting the N protein by ELISA from serum samples of healthcare workers affected by COVID-19 was also carried out. High anti-SARS-CoV-2 N-specific IgG Ab titers were detected in all except two of healthcare workers affected by COVID-19 (Figure 2a).

### 2.3. Identification of Linear B Cell Epitopes from Conserved Regions between SARS-CoV-2 and HCoV-OC43 N Proteins

**Figure 3:**
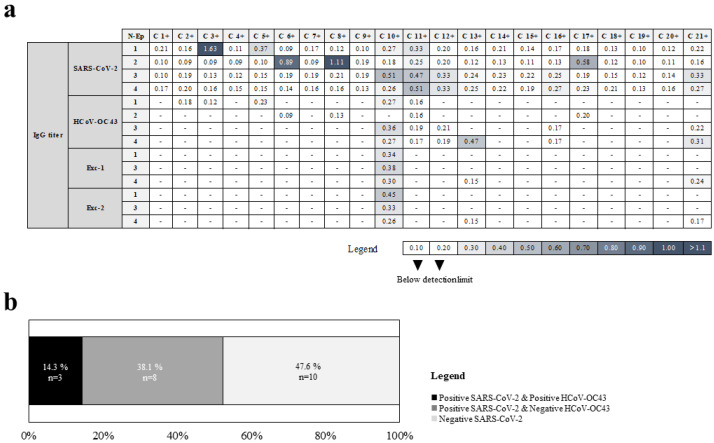
Reactivity against peptides candidates from SARS-CoV-2 and HCoV-OC43 N proteins in healthcare workers with COVID-19 determined by ELISA assays. (**a**) Heatmap with OD450nm readings for each sample. Cutoff for negative binding was established at OD450 = 0.2. (**b**) Frequency of cross-reacting serum samples among all tested serum samples in healthcare workers with COVID-19.

Next, synthetic peptides that mimic the four hypothetical antigenic conserved regions between SARS-CoV-2 and HCoV-OC43 N proteins were analyzed by ELISA assays in the 21 subjects. Among the coronaviruses analyzed, HCoV-OC43 was selected because it has an intermediate range of changes compared to SARS-CoV-2. Slightly more than half of the healthcare workers affected by COVID-19 (12/21, 57%) showed reactivity with any of the 4 SARS-CoV-2 N peptides tested (Figure 3a, Table 1). Each of the four N-derived peptides was recognized by IgGs from 4 to 5 individuals (Figure 3a, Table 1). Some healthcare workers with COVID-19 showed reactivity with two (C 12+, C 16+, and C 21+ individuals), three (C 10+), or all of SARS-CoV-2 peptides analyzed (C 11+) (Figure 3a, Table 1). These data demonstrate that the four SARS-CoV-2 N proteins regions tested are epitopes for B cells from multiple subjects.

Moreover, three sera from COVID-19-affected healthcare workers recognized HCoV-OC43 N peptides: C 13+ and C 21+, which were positive with the N-Ep4 peptide, and C 10+ sample with N-Ep1, N-Ep3, and N-Ep4 peptides, representing 14.3% of subjects (Figure 3b). These data demonstrate that three out of four HCoV-OC43 N proteins regions tested are recognized by specific Abs.

### 2.4. Serologic Reactivity of Anti-S and -N IgG Abs in a Pre-COVID-19 Cohort from 2016

Similar to SARS-CoV-2-infected subjects, antibody responses against SARS-CoV-2 in 40 serum samples obtained prior to the COVID-19 pandemic were estimated for the presence of anti-SARS-CoV-2 IgG Abs by ELISA. Very low IgG responses against SARS-CoV-2 S protein were detected in three serum samples (Figure 2b). Moreover, no IgG responses against SARS-CoV-2 S protein were detected in the other 37 pre-COVID-19 samples (Figure 2b). Finally, Anti-SARS-CoV-2 N-specific IgG Ab titers (ranged between 200 and 3200) were detected in 14 pre-COVID-19 samples in the ELISA assay (Figure 2b). The other 26 serum samples shown no (21) or very low (5) IgG responses against SARS-CoV-2 S protein (Figure 2b).

### 2.5. Identification of Cross-Reactive Linear B Cell Epitopes between SARS-CoV-2 and HCoV-OC43 N Proteins

**Figure 4:**
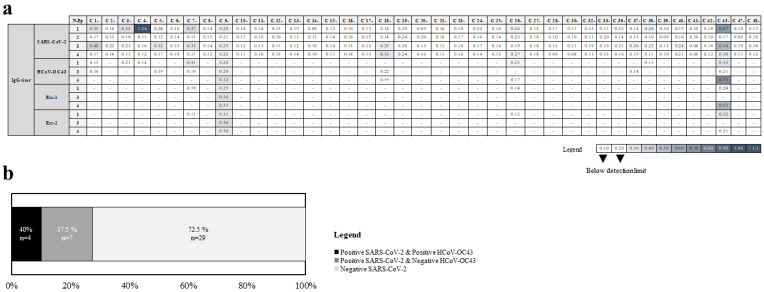
Reactivity against peptide candidates from SARS-CoV-2 and HCoV-OC43 N proteins in pre-COVID-19 cohort determined by ELISA assays. (**a**) Heatmap with OD450nm readings for each sample. Cutoff for negative binding was established at OD450 = 0.2. (**b**) Frequency of cross-reacting serum samples among all tested serum samples in the pre-COVID-19 cohort.

Dual recognition of SARS-CoV-2 and HCoV-OC43 N proteins by serum samples from healthcare workers affected by COVID-19 not demonstrating cross-reactivity between B cell epitopes because previous seasonal HCoV infections cannot be ruled out in these individuals. Thus, different Abs against the same conserved N protein regions may have been secreted by different B cell clonotypes in each individual. Synthetic peptides that mimic the four hypothetical antigenic conserved regions between HCoV-OC43 N and SARS-CoV-2 N proteins were analyzed by ELISA assays in the 40 pre-COVID-19 samples. Four sera (10%) from pre-pandemic samples recognized HCoV-OC43 N peptides: C 7-, and C 26-, which were positive with the N-Ep1 peptide, C 43- sample with N-Ep1, and N-Ep4 peptides, and C 9- sample that recognized N-Ep1, N-Ep3, and N-Ep4 peptides (Figure 4a, Table 2). Interestingly, identical recognition of HCoV-OC43 N peptides was obtained with their homologues from SARS-CoV-2 N protein in the four pre-COVID-19 samples in 40% of subjects (Figure 4b, Table 2).

For a more detailed analysis of the three cross-reactivities detected between HCoV-OC43 N and SARS-CoV-2 N protein epitopes, two consensus peptide sequences conserved between HCoVs were also tested (Figure 1c). All four possible patterns were detected: no reactivity with Exc-1 and Exc-2 peptides of Exc1 epitope (C 7-, and C 26- samples), reactivity with both Exc-1 and Exc-2 peptides of the three Exc epitopes from C 9- sample, reactivity with Exc-1 (but not Exc-2) peptide of Exc4 epitope from C 43- sample, and reactivity with Exc-2 (but not Exc-1) peptide of Exc1 epitope from this last pre-COVID-19 sample (Table 2), indicating a complex topology of epitope-Ab interaction.

## 3. Discussion

Identification of pathogen epitopes contributes to a better understanding of the antiviral immune response and rational vaccine design. In COVID-19, a new zoonotic disease which has resulted in pandemic, relevant efforts carried out in recent months have identified hundreds of epitope peptides recognized by CD8^+^ cytotoxic, and CD4^+^ helper T cells [2,3,4]. Furthermore, in the determination of B cell epitopes against this coronavirus, two studies, both using massive peptide libraries, identified multiple regions of different SARS-CoV-2 proteins as recognized by antibodies [6,13]. In these and other studies, different cross-reactive antibodies between SARS-CoV-2 and other HCoVs targeted against the S, but not N, protein were also identified [6,13]. In the current study, we performed a bioinformatic-and IEDB meta-analysis to identity linear B cell epitopes in the SARS-CoV-2 proteome that are conserved in HCoVs. Discontinuous B-cell epitopes are also very relevant in the humoral immune response, but their experimental validation is laborious and not subject to screening. As in our analysis, multiple hypothetical epitopes were predicted in different regions of the four structural viral proteins; we focused on those highly conserved between betacoronavirus for its interesting functional relevance in the epidemiological and evolutionary context of the current pandemic. Only four selected candidate peptide sequences, all in the N protein, satisfied the stringent antigenic and conservation criteria demanded. All were recognized as B cell epitopes by antibodies from multiple SARS-CoV-2^+^ subjects, showing the quality of the bioinformatic analysis carried out.

Five of these individuals even elicited antibodies against several of the epitopes analyzed. Similar functional analysis with the homologous peptides of HCoV-OC43 N protein showed that three of these four peptides tested were also B lymphocyte epitopes against this other coronavirus in different SARS-CoV-infected subjects. As these individuals may have been previously exposed to other coronaviruses, we cannot formally demonstrate cross-reactivity; even so, this reactivity of homologous regions with SARS-CoV-2 could be relevant. In addition, a significant fraction (4/40) of samples of the pre-COVID-19 cohort from 2016 recognized homologs peptides from both HCoV-OC43 and SARS-CoV-2 N proteins and, thus, properly, these four individuals generated HCoV-OC43 Abs cross-reactive with SARS-CoV-2 N protein. Overall, these epitopes, to our knowledge, are the first viral sequences identified as HCoV cross-reactive B epitopes against SARS-CoV-2 N protein. The fact that anti-SARS-CoV-2 N-specific IgG Ab titers were detected as well in other 10 pre-COVID-19 individuals different to C 7-, C 9-, C26-, and C43- samples indicates that cross-reactivity with other coronaviruses not analyzed in this study and/or non-linear, discontinuous epitopes conserved between SARS-CoV-2 and HCoV-OC43 must exist.

Moreover, the cross-reactivity of anti-S antibodies developed for COVID-19 patients to the HCoV homolog proteins is infrequent and weak [6,9,10,11,12,13], and very likely limited to some similarity between discontinuous epitopes. Conversely, the highest conservation of the B-cell linear epitopic peptides of N appears to be bi-directionally recognized between SARS-CoV-2 and HCoV antisera from our pre-pandemic samples. In contrast to S, N protein does not play direct role in viral attachment to epithelial cells of the upper and lower respiratory tracts so that anti-N antibodies are neither neutralizing nor protective against SARS-CoV in animal models [28]. In addition, this protein is ten-fold more abundant than S protein in the virion [29], very immunogenic [30], and extraordinarily enriched in flexible loops carrying validated and predicted B-cell epitopes [31]. Moreover, N protein is highly conserved among coronaviruses, probably due to high selective pressure to maintain numerous interactions with RNA in addition to viral and host proteins required by its role in virulence [32]. Conservation of humoral N epitopes between coronavirus may favor immune escape since its decoy effect may benefit the infection in individuals that experienced former seroconversion against the nucleocapsid of endemic HCoVs. This effect has been observed for anti-epitopes present in porcine circovirus [33] and lentivirus [34] antigens. Thus, the interpretation of the role of N humoral cross-recognition detected in the current report in the context of COVID-19 is not straightforward, but the coronavirus N proteins could elicit a misleading humoral cross-response, a hypothesis that should be analyzed in future studies.

## 4. Materials and Methods

### 4.1. Sequence Bioinformatics

Structural coronavirus reference sequences were downloaded from NCBI database SARS-CoV (Accession: NC_004718), SARS-CoV-2 (NC_045512), MERS (NC_019843), HCoV-HKU1 (NC_006577) HCoV-229E (NC_002645), HCoV-NL63 (NC_005831), and HCoV-OC43 (NC_006213) from National Center for Biotechnology Information database. Sequences were aligned by Muscle 3.8 [35]. Linear B-cell epitopes were predicted in SARS-CoV-2 structural sequences as described elsewhere [31]. Transmembrane helices were predicted with Phobius [36] and not considered to define B-cell epitopes since they are buried, i.e., not accessible, in the membrane. B-cell assayed epitopes were downloaded from Immune Epitope DataBase (IEDB) [20], using the following search words: “Positive epitopes”; assays, “B-cell”, type of epitope, “linear”; and organism, “SARS-CoV“ or “SARS-CoV-2”, according to the score. The experimental score is the agreement (0-1) of the epitope identification respect to the total number of contributing references for that protein for either SARS-CoV or SARS-CoV2. The computational score is the agreement of eight algorithms that predict linear B-epitopes as previously calculated [31].

### 4.2. Subject Details

Twenty-one healthcare workers affected by COVID-19 at the Hospital 12 de Octubre, Madrid, Spain, were invited to participate in the study after providing informed consent. Patients were identified as COVID-19 positive through serologic testing (20 individuals) or via clinical diagnosis based on symptomology followed by a positive SARS-CoV-2 RT-PCR (one patient). The study was approved by the Ethics Commitee of the Hospital 12 de Octubre, Madrid, Spain. Furthermore, 40 pre-COVID-19 samples obtained in 2016 were included in the study under the Ethics Committee of the ISCIII.

### 4.3. S and N Protein and Peptide Specific ELISAs

Recombinant S protein was produced by transient transfection of FreeStyle 293F cells (Thermo Fisher, Waltham, MA, USA) using polyethylenimine and a plasmid coding for an HexaPro derived construct [37] that includes the D614G substitution. The S ectodomain was purified from filtered cell supernatants using HisTrap™ Excel columns (CYTIVA) and subjected to an additional purification step by size-exclusion chromatography using a Superose 6 10/300 column (CYTIVA). Recombinant N protein with C-terminal His-tag, derived from the transfected human HEK293 cells was purchased from RayBiontech (Ref 230-30164-100). For indirect ELISAs, plates were coated with 100 ng of recombinant protein, and then processed as described previously with two-fold dilutions of the serum samples from 1:100 to 1:204800. Titers were defined as the highest dilution of serum giving an OD450 at least 0.5 above wells that were processed in the absence of serum. In addition, 96-Well Clear Flat Bottom Polystyrene High Bind Microplate were used for all ELISA assays (Corning, Ref 159018).

For N protein peptide ELISAs, peptides corresponding to the indicated epitopes were synthesized by ProteoGenix (Schiltigheim, France). Lyophilized peptides were resuspended in sterile PBS to a concentration of 1 mg/mL to create stock solutions. The stock solutions were diluted to a final concentration of 1 µg/mL in PBS, and 100 µL of this solution was used to coat ELISA plates, to achieve 100 ng per well. ELISAs were performed as described above with 1:100 dilutions of the serum samples. Samples were defined as positive if the OD450 was at least 0.2 above wells that were processed in the absence of serum.

## Figures and Tables

**Table 1:** Summary of reactivity against peptides candidates with functional reactivity from SARS-CoV-2 and HCoV-OC43 N proteins in healthcare workers with COVID-19 determined by ELISA assays. ^a^ The numbers indicate the positive ELISA assay from peptides indicated in Figure 1c.

Sample	Reactivity with N-Ep Peptides from	
	SARS2	OC43
C 2+	N-Ep1 ^a^	
C 3+	N-Ep1	
C 5+	N-Ep1	
C 6+	N-Ep2	
C 8+	N-Ep2	
C 10+	N-Ep1, N-Ep3, N-Ep4	N-Ep1, N-Ep3, N-Ep4
C 11+	N-Ep1, N-Ep2, N-Ep3, N-Ep4	
C 12+	N-Ep3, N-Ep4	
C 13+	N-Ep4	N-Ep4
C 16+	N-Ep3, N-Ep4	
C 17+	N-Ep2	
C 21+	N-Ep3, N-Ep4	N-Ep4

**Table 2:** Summary of reactivity against peptides candidates with functional reactivity from HCoV-OC43 and SARS-CoV-2 N proteins in pre-COVID-19 cohort determined by ELISA assays. ^a^ The numbers indicate the positive ELISA assay from peptides indicated in Figure 1c.

Sample	Reactivity with N-Ep Peptides from			
	OC43	SARS-CoV-2	Exc-1	Exc-2
C 7-	N-Ep1 ^a^	N-Ep1		
C 9-	N-Ep1, N-Ep3, N-Ep4	N-Ep1, N-Ep3, N-Ep4	1,3,4	1,3,4
C 26-	N-Ep1	N-Ep1		
C 43-	N-Ep1, N-Ep4	N-Ep1, N-Ep4	4	1

## Data Availability

All data are included in the manuscript.
